# Social interactions lead to motility-induced phase separation in fire ants

**DOI:** 10.1038/s41467-022-34181-0

**Published:** 2022-11-07

**Authors:** Caleb Anderson, Alberto Fernandez-Nieves

**Affiliations:** 1 Department of Condensed Matter Physics, University of Barcelona, 08028 Barcelona, Spain; 2 School of Physics, Georgia Institute of Technology, Atlanta, GA 30332 USA; 3 ICREA-Institució Catalana de Recerca i Estudis Avançats, 08010 Barcelona, Spain; 4 Institute for Complex Systems (UBICS), University of Barcelona, 08028 Barcelona, Spain

**Keywords:** Phase transitions and critical phenomena, Soft materials, Condensed-matter physics

## Abstract

Collections of fire ants are a form of active matter, as the ants use their internal metabolism to self-propel. In the absence of aligning interactions, theory and simulations predict that active matter with spatially dependent motility can undergo motility-induced phase separation. However, so far in experiments, the motility effects that drive this process have come from either crowding or an external parameter. Though fire ants are social insects that communicate and cooperate in nontrivial ways, we show that the effect of their interactions can also be understood within the framework of motility-induced phase separation. In this context, the slowing down of ants when they approach each other results in an effective attraction that can lead to space-filling clusters and an eventual formation of dynamical heterogeneities. These results illustrate that motility-induced phase separation can provide a unifying framework to rationalize the behavior of a wide variety of active matter systems.

## Abstract (web summary)

Some active matter systems as they evolve, can be characterized by spatially varying density, with some regions that are dense and immobile, and other regions with lower density that accommodate most mobile particles. The authors show that this phenomenon can also be observed as an effect of the social interactions between fire ants.

## Introduction

Active matter systems, which include flocks of birds^1,2^, swarms of bacteria^3,4^, and collections of fire ants^5–7^, are far from equilibrium because they violate the principle of detailed balance^8,9^. This principle, reflecting the equal likelihood for a process at the microscopic level to occur in one and the opposite direction, is a hallmark underlying all of equilibrium statistical mechanics^10^. In contrast, ants convert energy stored in adenosine triphosphate (ATP) into kinetic energy, which is then dissipated via frictional forces, but there is no reverse process in which the ants can convert their kinetic energy into stored chemical energy. Despite this significant difference, active matter is often best understood in analogy to equilibrium systems. For example, systems of self-propelled particles with local aligning interactions undergo the equivalent of a phase transition, which is a feature of some equilibrium systems, from a disordered phase to a polar-ordered phase with the control parameters of noise and density^11,12^. This phase transition results in traveling flocks and herds^13^ in living systems and sometimes, in phase coexistence^14,15^. More recently, interest has grown in active systems with particles that have negligible aligning interactions but have spatially varying self-propulsion speeds, which could arise due to crowding, particle interactions, or the influence of external parameters, such as chemical gradients^16^ or variable lighting levels for photosensitive synthetic active particles^17,18^.

At low densities, it is theorized that if a spatially dependent motility can result in a spatially varying density, the system can be mapped onto the behavior of an equilibrium system in the presence of an effective potential^19,20^. If this motility-induced effective potential depends strongly on local density, theory and simulations show that the system can undergo motility-induced phase separation (MIPS) into regions that are maximally dense, sometimes crystalline, and regions with much lower density and more mobile particles^19–21^. Such phase separation has been verified in bacteria exposed to chemical gradients^16^, and with Quincke rollers^22^ or hard Janus particles interacting via collisions^23,24^. In each of these cases, the motility effects that drive MIPS have come from direct collisions or crowding, which restrict the motion of individual particles, or from an externally applied field, so it is currently unknown how broadly applicable these principles may be.

Here, we show that this framework can be extended to understand the behavior of fire ants, *Solenopsis invicta*, social insects known to communicate with each other through pheromones and physical contact^25,26^. We find that we can indeed describe their behavior using an effective motility-induced potential that leads to MIPS.

## Results

### Motility-induced effective potential

**Fig. 1:**
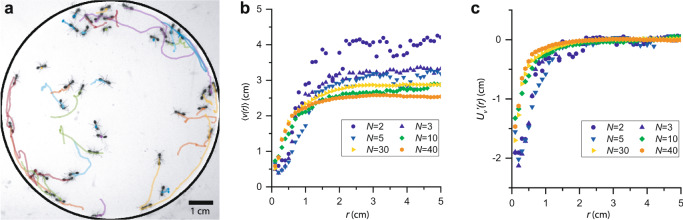
Motility-induced attraction between ants. **a** Forty fire ants confined in a 2D cell with their tracked trajectories over the previous 3 s shown as colored lines. The edge of the cell is outlined in black for clarity. **b** Average speed of the ants as a function of their center-to-center distance. **c** Motility-induced effective potential between pairs of ants, calculated from the average velocities for a given center-to-center distance.

We confine sterile female fire ants, which have an average length *l* = (3.5±0.7) mm, to a circular cell with a diameter *D* = (9.00 ± 0.02) cm and a height *h* = (1.6±0.1) mm, which is slightly larger than the height of an ant (see “Methods”). The ants are thus effectively confined to two dimensions. We place this cell in the plane perpendicular to the gravitational direction, image the ants from above, and track their positions as a function of time. For example, Fig. 1a shows an image of *N* = 40 ants in a cell, together with their trajectories over the previous 3 s, which are shown as colored lines. Notice that the ants that have the shortest trajectories in this figure, and therefore lowest speeds over the 3 s, tend to be near other ants. This is a typical result of their social interactions, which we quantify by measuring the average speed of an ant as a function of its distance from another ant. We sort the velocities of pairs of ants by their center-to-center distance, which we divide into 1 mm bins, ensuring that there are at least 20,000 measurements in each bin with 0.3 cm < *r* <7 cm, and take the average for each bin. The resulting <*v*(*r*)> for various *N* are shown in Fig. 1b. This figure can be understood by considering a reference ant at *r* = 0 and a second ant a given distance, *r*, away from the reference ant. Figure 1b then shows the average speed at which we measure the second ant to be moving. Notice that <*v*(*r*)> depends on *N* because this second ant is free to interact with ants in the cell other than the reference ant. Regardless, for all *N*, we find that the ants, on average, slow down as they approach each other. To account for the effect of the ants’ spatially dependent motility, we consider a continuity equation for the probability, *P*, that an ant has position **r**, and orientation *θ*, if the ant moves in the direction it is facing^19,27,28^:

1 $$\dot{P}\left({{{{{\bf{r}}}}}},\theta \right)=-\vec{\nabla }\cdot \left[P\left({{{{{\bf{r}}}}}},\theta \right)v\left({{{{{\bf{r}}}}}}\right)\hat{{{{{{\bf{u}}}}}}}\right]+\varTheta \left(P\left({{{{{\bf{r}}}}}},\theta \right)\right)$$

where $\hat{{{{{{\bf{u}}}}}}}={{\cos }}\left(\theta \right)\hat{{{{{{\bf{x}}}}}}}+{{\sin }}\left(\theta \right)\hat{{{{{{\bf{y}}}}}}}$ and $\Theta \left(P\left({{{{{\bf{r}}}}}},\theta \right)\right)$ controls the change in the orientation of the ants. In most systems, this function is taken as $\Theta \left(P\left({{{{{\bf{r}}}}}},\theta \right)\right)={D}_{{{\theta }}}\left({{{{{\bf{r}}}}}}\right)\frac{{\partial }^{2}}{\partial {\theta }^{2}}P\left({{{{{\bf{r}}}}}},\theta \right),$ where *D*_*θ*_ (**r**) is a rotational diffusion constant that depends on location. The rate of change of *P* thus depends on the divergence of the probability current, where the minus sign indicates that an outward flux of the probability current decreases *P*. The $\varTheta \left(P\left({{{{{\bf{r}}}}}},\theta \right)\right)$ term then acts as a source or sink for $P\left({{{{{\bf{r}}}}}},\theta \right)$. Interestingly, for isotropic processes, in which all relative orientations are equally likely and independent of position, $P\left({{{{{\bf{r}}}}}},\theta \right)=P({{{{{\bf{r}}}}}})/(2\pi )$, a steady state solution is $P\left({{{{{\bf{r}}}}}}\right)\propto v{\left({{{{{\bf{r}}}}}}\right)}^{-1}$, suggesting that the ants are more likely to be in regions in which they move more slowly so that $P\left({{{{{\bf{r}}}}}}\right)v({{{{{\bf{r}}}}}})$ is constant across the cell.

To physically think of this, we consider a model equilibrium system consisting of a single particle in contact with a thermal reservoir and subjected to an external potential *U*(*r*). The probability of finding this particle with a given position is $P\left(r\right)\propto {{\exp }}\left(\frac{-U\left(r\right)}{{kT}}\right)$, where *kT* is the thermal energy. For this equilibrium particle to have the same probability distribution as that predicted by the continuity equation above in steady state, the model potential would need to be ${U}_{v}\left(r\right)={kT}{{{{{\rm{ln}}}}}}\left(v\left(r\right)/{v}_{0}\right)$, where we have chosen as the scale for speed the average speed, *v*_0_, of ants with center-to-center distances 4 cm ≤ *r* ≤ 6 cm.

In our case, since the ants’ motion is not related to thermal fluctuations, we have no convincing measure of an effective *kT*. However, we can still see the effect of the motility by examining the shape of the motility-induced effective potential, which we define as ${U}_{v}^{{\prime} }={{{{{\rm{ln}}}}}}\left( < v\left(r\right) > /{v}_{0}\right)$, neglecting any prefactors. We find that ${U}_{v}^{{\prime} }$ increases as *r* increases, as shown in Fig. 1c. This indicates that the effect of social interactions is equivalent to an attraction, since the interaction force $F=-d{U}_{v}^{{\prime} }/{dr}$ is negative and thus along the $-\hat{{{{{{\boldsymbol{r}}}}}}}$ direction. We note that one of the effects that is known to disrupt this analogy is the presence of aligning interactions, which are common in many active matter systems^17^. In the presence of aligning interactions, the relative orientations between particles are no longer independent of position and the solution to the continuity equation (1) breaks down. Though we do find some evidence of alignment between the ants in our experiments, these are only relevant for very small center-to-center distances (see “Methods”). Importantly, because the ants are, on average, barely moving when they have small center-to-center distances, alignment does not seem to disrupt the effective attraction.

Notice that our measurement of ${{U}{\prime}}_{v}$ for *N* = 2 implies that attractive forces between a pair of ants appear localized to within about 1.3 *cm* ≈ 3.5*l*, which is approximately the distance at which fire ants are thought to be able to detect one another^29^, and thus begin to interact.

### Potential of mean force

We now check for motility-induced attraction by independently measuring the probability distribution for the ants’ pair distances and calculating the potential of mean force (PMF) between two ants. The PMF is the pair-potential associated with the average force between two particles, given all possible configurations of all other particles in the system. It is a powerful notion often used to study equilibrium many-body systems, such as colloidal suspensions^30,31^. We first measure the pair distribution function, *g*(*r*), for the ants, which compares the probability of finding a pair of ants with a given separation, *P*(*r*), to the probability of finding a pair of ants very far apart, *P* (*r* → *∞*). In a system far from the boundaries, $g\left(r\right)=\frac{P(r)}{P(r\to \infty )}=\frac{1}{\rho }\frac{N\left(r\right)}{2\pi r{\Delta }r}$, where *N*(*r*) is the number of ants in an annulus of thickness *Δr* centered at *r*, and *ρ* = *N*/*A*, with *A* the available area, is the particle density. Whereas $\mathop{{{{{{\rm{lim}}}}}}}\limits_{r\to \infty }N(r)\to \infty$, $\mathop{{{{{{\rm{lim}}}}}}}\limits_{r\to \infty }g(r)\to 1$. The restrictive dimensions of our cell requires modifying our calculation because the possible circumference is no longer $2\pi r$ when the ants are near the cell walls. However, we can correct for this geometrically (see “Methods”). For an equilibrium system, we can use $g(r)$ to obtain the PMF, because by definition, $g\left(r\right)={{\exp }}\left(-\frac{{U}_{p}\left(r\right)}{{kT}}\right)$, where ${U}_{p}(r)$ is the PMF. For the ants, we define ${U}_{p}=-{{{{{\rm{ln}}}}}}\left(g\left(r\right)\right)$, neglecting prefactors. Note that the PMF is distinct from the more familiar pair interaction potential, as it includes contributions from forces intermediated by particles other than the observed pair. The result is that *U*_*p*_ varies with *N* for the same reason ${U}^{\prime}_{v}$ does.

**Fig. 2:**
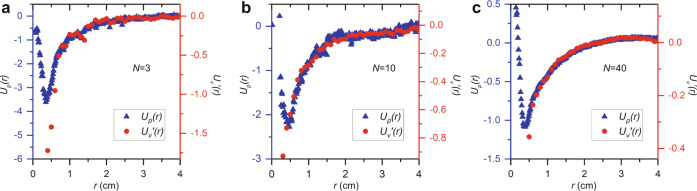
Comparison of effective potentials. **a**–**c** The PMF (left axis, blue triangles) and motility-induced effective potential (right axis, red circles) for trials with *N* = 3, 10, and 40 ants, respectively.

Remarkably, at moderate to high pair distances, the shape of the ants’ *U*_*p*_ and ${U}^{\prime}_{v}$ agree very well, as shown in Fig. 2a–c, for *N* = 3, 10, and 40 ants, respectively. On the one hand, this indicates that the motility-induced effective potential can be physically important in dry active systems, such as crowds of creatures. On the other hand, notice that for *r* < 4 mm, *U*_*p*_ is effectively repulsive, because the interaction force *F* = −*dU*_*p*_/*dr* is positive and thus along $\hat{r}$. The source of this repulsion is a combination of excluded area caused by the shape of the ants’ bodies and a tendency of the ants to avoid crowding each other’s legs. Since these effects are not related to the ants’ motility, the repulsion is not present in their motility-induced potential, ${U}_{v}^{{\prime} }(r)$.

### Clustering and phase separation

**Fig. 3:**
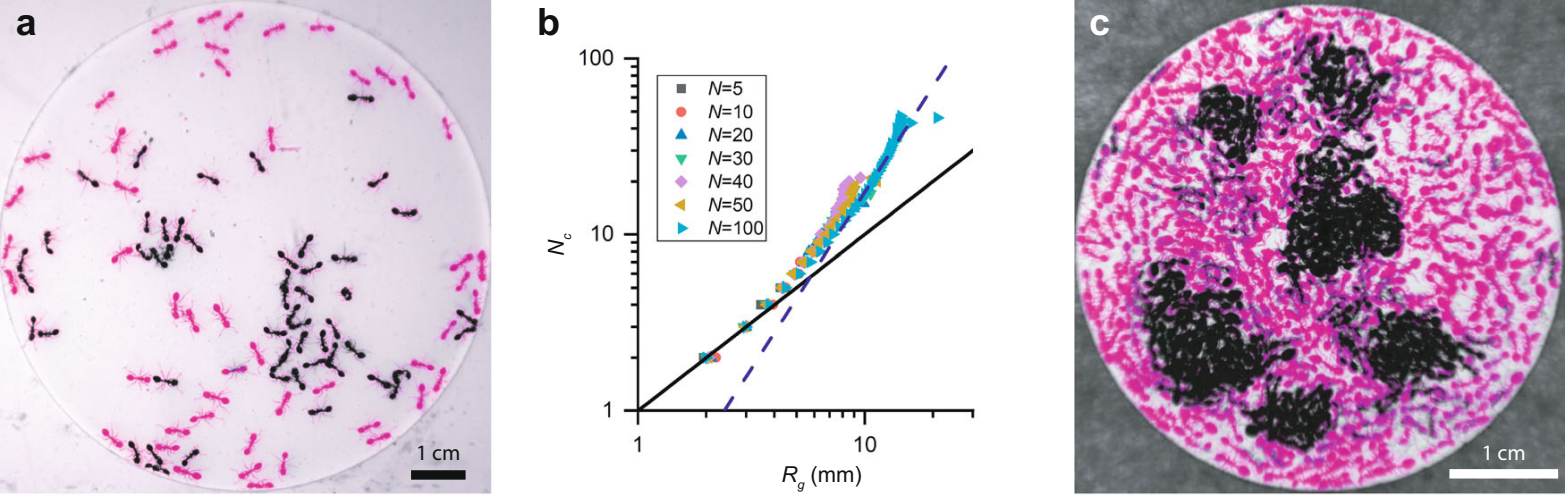
Clustering and phase separation. **a** False color image of 100 ants in a cell with *D* = 9.00 cm. Stationary ants are shown in black and moving ants are shown in magenta. **b** Number of ants in a cluster versus its radius of gyration for various *N*. The black line shows *N*_*c*_ ∝ *R*_*g*_ and the blue dashed line shows ${N}_{c}\propto {R}_{g}^{2}$. **c** False color image of about 625 ants in a cell with *D* = 4.50 cm. Ants that remain stationary over thirty seconds are shown in black and ants that move over thirty seconds are shown in magenta.

The effective attractive force between the ants leads to the formation of clusters of nearly stationary ants. At low densities, the system exhibits short-lived clusters of non-moving ants, like the ones shown in the false color image of *N* = 100 ants in Fig. 3a. In this figure, the ants that are currently near-stationary, which we define as having an average speed below 1 mm/s between two frames, are indicated in black, and the rest of the ants are shown in magenta. Notice that most of the stationary ants are in clusters, including a large cluster consisting of more than 20 ants. All the stationary ants in this frame began moving within the next 30 s. These short-lived clusters can also be seen clearly in Sup. Movie 1. Ants in the wild often interact for the purpose of allogrooming or communication^25^. We notice that the ants in these experiments often probe their surroundings with rapid antenna motion when they first join a cluster or interact with another ant, but this antenna motion, along with all other slight body movements tends to stop within several seconds after an ant joins a cluster. We therefore believe that the main biological function of the ants’ interactions in these experiments is either energy conservation or aggregation promotion.

We group the stationary ants into clusters using a threshold distance of 7 mm ≈ 2*l* on the center-to-center distance between ants and calculate the average radius of gyration, *R*_*g*_, for clusters made up of different numbers of ants, *N*_*c*_ (see “Methods”). Our results in Fig. 3b show that large clusters tend to fill space, ${N}_{c}\propto {R}_{g}^{2}$, as demonstrated by the dashed blue line. These space-filling clusters are reminiscent of clusters seen in other active systems^24^, including aggregations naturally formed by other species of ants^32^ and cockroaches^33^. At these low densities, we find that the ants in the center of the clusters are free to activate and leave. As a result, the size of the clusters fluctuates quickly and does not continuously grow in time (see “Methods”).

For high densities, the motility effects in active systems can often result in an instability, in which local fluctuations in the density can decrease the local motility. The local decrease in motility can cause a local increase in $\left|{U}_{v}\right|$, which in turn causes the density to continue to increase. In this case, $\rho ({{{{{\bf{r}}}}}})\propto P\left({{{{{\bf{r}}}}}}\right)\propto {{\exp }}\left(-\frac{{U}_{v}\left({{{{{\bf{r}}}}}}\right)}{{kT}}\right)$. This runaway process results in motility-induced phase separation, in which the system separates into regions with stationary particles at very high density and regions of moving particles at much lower density.

To test this expectation, we switch to smaller confining cells with *D* = (4.50 ± 0.02) cm, so that we can reach high enough *ρ*, and observe the ants’ behavior. At these densities, we are unable to track individuals. However, we observe that the ants clearly begin to form spatial heterogeneities that last hundreds of seconds (see “Methods”). These heterogeneities are visible as dense clusters of stationary ants surrounded by a background of more quickly moving ants, as shown in Sup. Movie 2; the observed spatial heterogeneities thus also correspond to dynamic heterogeneities. Figure 3c shows a typical frame captured during an experiment with *N* ≈ 625 ants. In this false color image, pixels that remain dark for the next 30 s are shown in black and pixels that were dark when this frame was captured but then changed to light anytime over the next 30 s are shown in magenta. This makes it clear that many of the densest regions in the cell are filled with stationary ants, in agreement with the MIPS framework.

Unlike in the clusters formed when the ants are confined at low density, at high density, the ants on the interior of the clusters are no longer free to leave the clusters. While the clusters still melt and reform over long times, we find that the clusters coarsen such that their length scale grows in tentative agreement with $L\propto {t}^{\frac{1}{3}}$, as expected for MIPS and more generally seen in spinodal decomposition processes^20,34^ (see “Methods”).

### Departure from MIPS

**Fig. 4:**
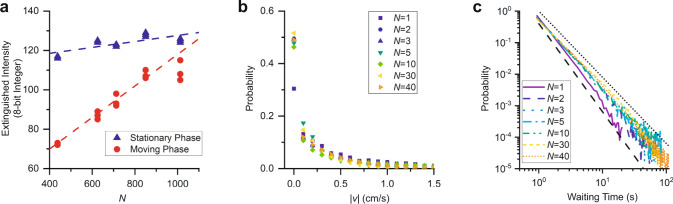
Departure from equilibrium analogy. **a** Measurements of the intensity of the light extinguished by the stationary clusters (blue triangles) and the actively moving phase (red circles) for various numbers of ants contained in *D* = 4.50 cm cells. The dashed lines are guides to the eye to show that both densities are increasing. **b** The speed distributions for various numbers of ants in *D* = 9.00 cm cells. After the addition of a second ant, further additions do not appreciably change the speed distributions. **c** The probability distributions of waiting times between ceasing and beginning motion. The distributions agree with power laws (dashed and dotted lines).

While MIPS explains the existence of dynamic heterogeneities in the ants at high densities, the social behavior of the ants causes a surprising departure from traditional phase separation. In equilibrium phase coexistence, a change in global density changes the proportions of the coexisting phases, but the density of each phase remains unchanged. Based on this expectation, we look at the details of the observed phase coexistence in our fire-ant crowds and measure the difference in light extinction, detected as an unsigned 8-bit integer, between an empty cell and each of the phases, which serves as a proxy for the number density of the stationary and moving phases of the ant-crowds (see “Methods”). We find that increasing the number of ants in the cell results in an increase in the density of both phases, as shown by the light extinguished by each phase in Fig. 4a, in which blue triangles represent the density of the stationary phase and red circles represent the density of the moving phase. The fact that the density of the coexistent phases is not constant reflects a density-dependent vapor pressure, which is a known effect in multicomponent liquid-liquid phase separation^35^, like those common in intracellular environments,^36^ but that has not been seen in single component liquid-gas phase separation or MIPS.

This departure from the equilibrium analogy must result from the specifics of how ants socially interact. To consider the source of this difference, we determine the speed distributions of the ants in our tracking experiments with various *N*, see Fig. 4b. We find there is a noticeable difference in the speed distribution of single ants compared to the speed distributions with more ants. While a single ant spends only about 30% of its time moving slower than 1 mm/s, for *N* > 1, the ants spend about 50% of their time moving slower than 1 mm/s. However, adding more ants to the cell after the second ant does not appreciably change the speed distribution. The ants thus have a predisposition to spend about half their time moving and about half of their time stationary, regardless of density, provided they have at least one companion in the cell. This is at odds with what one might expect, as increasing the number of ants in the cell would typically decrease the speed of the ants, given that there would a priori be more locations in the cell where the ants could be slowed down due to their interactions with other ants. However, our finding agrees with previous experiments with *Solenopsis invicta* that show that the ants begin to avoid social interactions as the density increases so that their rate of contact remains unchanged, regardless of global density^29^.

To further illustrate that the social interactions of the ants remain unchanged for *N* > 1, we examine the waiting times of the ants, which we define as the time intervals that the ants remain stationary before they start moving again. Figure 4c shows that the ants do not exhibit a characteristic timescale for these waiting times. Instead, we find that the probability distribution for these waiting times agrees with a power law distribution. For *N* = 1, this power law has an exponent of *γ* = −2.8 (dashed line in Fig. 4c). When one more ant is added, this exponent decreases to *γ* = −2.1 (dotted line in Fig. 4c), indicating that the ants are now much more likely to have longer waiting times as a result of their interactions. However, adding more ants has no effect on the observed exponent, supporting the idea that the ants prefer a certain amount of social interaction, regardless of *N*.

Our findings indicate that an ant has a set fraction of time that it prefers to spend moving. If we further assume that the ants do not coordinate the timing of their activity, then, at any given moment, approximately the same proportion of ants will be moving. If the proportion of moving ants is fixed by the ants’ social behavior, then the densities of the two phases must change with global density, which is indeed what we find experimentally.

## Discussion

At low densities, pairs of ants interact via complicated social interactions that we can summarize by examining their spatially dependent motility. When we increase the density of ants in the cell, we find that the strength of these interactions begins to decrease, due to interactions with other ants in the cell. This is consistent with the principle of local activation/long range inhibition that governs many aspects of social insect behavior^32,37^. In this case, an ants’ interactions with other ants in the cell tend to decrease the likelihood of interactions with any particular ant. In terms of our model potential, the other ants in the cell exert a force on any given ant that points radially away from a reference ant.

Aggregation in biological systems is known to have evolutionary advantages^38^ and remains an active area of study with a variety of well-known theoretical models^39,40^, including some successful models that propose a density-dependent motility^41,42^ and bear a striking resemblance to the formalism of MIPS^19^. Under the right conditions, these models also predict stationary clusters with sharp edges embedded in an actively moving lower density background, consistent with the ant clusters we observe here. Our results thus provide an interesting case study where motility effects exist and result in the formation of essentially stationary clusters within a single crowded system.

Effective interaction potentials also have long history of being used to model animal behavior^43^. Recently, for example, success has been found in directly measuring effective interaction potentials of fish by detecting the acceleration of individuals as they approach each other^44,45^. Here, we have measured the interaction potential in a different manner that may be more appropriate to crowds of organisms, as it can easily capture multibody effects, and shown that the complex social interactions of ants can be rationalized in terms of existing theory for model active matter. Our results use the framework of MIPS to explain the behavior of multicellular organisms, showing that MIPS can indeed be useful in describing crowds of living creatures. This connection between social ants and active matter physics paves the way for developing new models to describe the behavior of crowds of other social creatures, such as humans. Modeling social creatures as equilibrium particles interacting with effective potentials may help predict the threshold levels at which crowd dynamics begin to slow down or stop. Taking the analogy of an effective potential further, we believe that certain room or hallway geometries could naturally be modeled as an external effective potential that causes clusters of people to gather in certain areas, allowing effective crowd control. However, despite the success of MIPS in describing what we observe with fire ants due social interactions, more work is needed to fully rationalize all of our results; this further work might include simulations to address our proposed source for a density-dependent “vapor pressure” and theory work to quantitatively obtain and compare both the motility potential and the PMF for various *N*, paying special attention to the physics behind the prefactors preceding their *r*-dependence.

## Methods

### Ant collection

We collect our fire ants from the wild in a vacant lot in Kennesaw, GA, (34°01'10.7“N 84°31'36.6“W) between March and October. To do this, we slick the walls of 5-gallon buckets with talcum powder to prevent ants from climbing out of them and then seek out the largest colonies available. We search for large above-ground mounds that are aggressively defended between 24 and 72 h after a heavy rain. We collect the ants along with the soil of their mound and subterranean tunnel system to a depth of about 20 cm and fill the buckets no more than two-thirds of the way to the rim. Once we have brought the ants to the lab, we leave the ants undisturbed in the buckets for one full day to allow the ants to form a tunnel system. Then we use a system of irrigation tubing to drip water into the soil at a rate of about 1 mL/s, flooding the buckets over the course of another two days. Dripping the water allows the ants to make their way to the surface of the soil without drowning. As the water level continues to rise past the surface of the soil, the ants form rafts on the water surface. We remove the rafts with a spoon, place them into large open Tupperware bins and, slick the walls of the bins with Polytetrafluoroethylene, also known as Fluon, a copolymer which keeps the ants from climbing out of the lidless bin. These bins make for convenient storage because they allow for easy removal of ants for experiments by keeping the ants away from soil.

Once in our care, the ants are given a continuous supply of high protein baby food, in particular a smooth purée containing chicken or turkey, and water so that they never run out. We supply water with a cotton ball that is resting in water, either in a shallow petri dish of water or as a stopper on a test tube filled with water. The ants are also given two overturned halves of a petri dish that have been blacked out, where they naturally store their eggs, winged males, and possibly queens. Outside of the blacked-out halves of petri dish, the ants are exposed to constant overhead lighting.

We calculate the average length of an ant by measuring the distance from the tip of the mandibles to the stinger for each of slightly more than 1000 ants from 10 different colonies. The average and standard deviation of our collected sterile females’ length are *l* = (3.5±0.7) mm.

We measure the average mass of our sterile females to be *m*_*ant*_ = (0.8 ± 0.1) mg. This average is calculated by weighing 5 samples of about 200 ants and the error contains the maximum and minimum average mass of the samples. This mass is compared to the total mass of a sample to calculate the number of ants we use in an experiment when *N* > 150 ants.

When we collect ants from the bins to use them in an experiment, it is convenient to chill the ants in a refrigerator to temporarily reduce their metabolism to ease the transfer. Chilling the ants for a short time does not harm them. However, the ants’ behavior changes after several weeks in captivity, even if the ants are fed and watered consistently. For this reason, we typically obtain new ants about once per month in the warmer months.

### Tracking

The cells are made by cutting a circle out of a thin sheet of acrylic and then sandwiching the acrylic between two pieces of glass. We place the acrylic on one sheet of glass, load the circular cell with *N* chilled ants, add the second piece of glass, place these cells horizontally, and back-light them through a sheet of light diffuser. We then quickly begin imaging the ants from above, so that the first image is taken no more than 10 s after the ants are added to the cell. After encountering the room-temperature glass of the cell, the chilled ants warm up and begin to wander the cell within several seconds.

After imaging the ants, we segment the images to detect the regions of pixels that correspond to ants and measure the area, *A*, and centroid, **r**, of each region. We also measure the orientation of each ant by first finding the ellipse that has the same second area moment as the group of pixels that we associate with an ant. We then calculate *θ* as the rotation angle of this ellipse; *θ* is the angle of the major axis of the ellipse relative to the x-axis of a lab frame.

By choosing to compare the ant to an ellipse, we have neglected polar orientation. We track the ants by minimizing the cost, *c*_*ij*_, between the ants in one frame and the ants in the next frame and adding the new ants to the existing tracks. In our case,

2 $${c}_{{ij}}={C}_{r}\left|\Delta {{{{{{\bf{r}}}}}}}_{{ij}}\right |+{C}_{\theta }\left|{{{\theta }}}_{{{{{{\rm{ij}}}}}}}\right |+{C}_{A}\left|\sqrt{{A}_{i}}-\sqrt{{A}_{j}}\right|$$

where *θ*_*ij*_ is the difference in orientation between the two ants, $\left|\Delta {{{{{{\bf{r}}}}}}}_{{ij}}\right |=\left|{{{{{{\bf{r}}}}}}}_{j}-{{{{{{\bf{r}}}}}}}_{i}\right|$ is the relative distance between ants, and *C*_*r*_, *C*_*θ*_, and *C*_*A*_ are constants set so that, on average, the distance between ants accounts for 90% of *c*_*ij*_. For computing efficiency, we first try a greedy solution to minimize the cost of connecting each of the ants, so that each track searches for the ant with the minimum cost to add to itself. Then, if two tracks both try to add the same ant, the track with the lower cost gets to keep the ant and the track with the higher cost must take its next choice. This process avoids the computationally expensive process of finding the global minimum cost. We begin tracking the ants at the beginning of the experiment and use all our tracking data to compute *θ*(*r*), *v*(*r*), *g*(*r*), waiting times, and *N*_*c*_. There is a transient state at the beginning of the experiments, in which the ants hardly interact, but this state is brief enough that it does not affect our averages.

### Alignment measurements

**Fig. 5:**
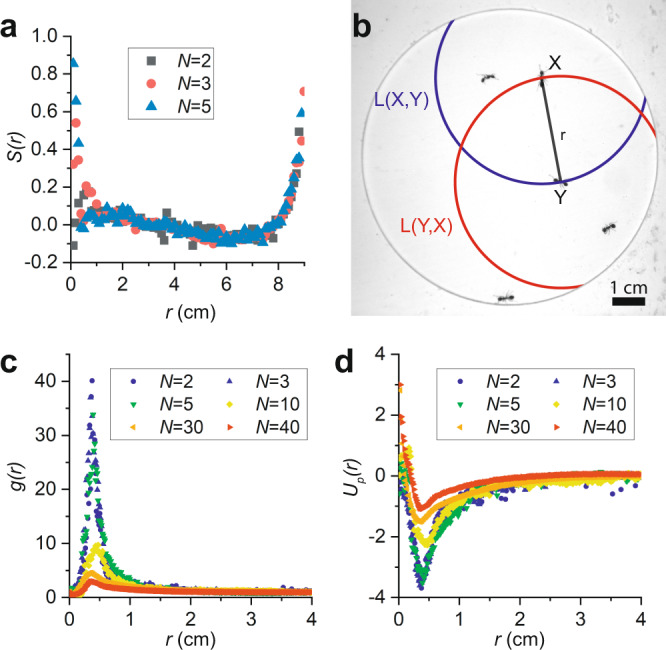
Alignment and pair correlations. **a** Alignment between pairs of ants demonstrated by the order parameter for pairs at various distances. **b** Illustration of the available arc length. The red line shows the available arc length, *L*(*Y,X*), for ant *Y* to be found a distance *r* away from ant *X* and the blue line shows *L*(*X,Y*). **c** Calculated pair correlation function after using our geometric correction for different *N*. **d** Potentials of Mean Force for different *N*.

We sort all measurements of *θ*_*ij*_, for ants in the same frame by center-to-center distance and measure the orientational order parameter, $S(r)=\langle 2{{{\cos }}}^{2}{\theta }_{{ij}}(r)-1\rangle,$in each bin. A value of *S* = 0 corresponds to completely disordered rods in 2D, while a value of *S* = 1 corresponds to completely ordered rods. Note that *S* can in principle take negative values; this occurs if the rods tend to align perpendicularly. In our experiments, we in fact observe that *S* takes slight negative values at intermediate *r*; this results from the alignment induced by the confining walls, which forces occurrence of configurations where the ants align perpendicular to each other along the boundary. Also recall that the ants are rather unlikely to be found very close together, which is evident from the relatively high values of *U*_*p*_ (*r*) when *r* is very small in Fig. 2. To aid our statistics then, we bin all pairs of ants from experiments with *N* = 2, 3, and 5 ants to find that the ants do align over short distances, as shown in Fig. 5a. Notice that the order parameter drops to near *S* = 0 steeply when *r* ≈ 4 mm ≈ *l*. When r approaches 9 cm, *S*(*r*) increases, since a pair of ants at this separation are at opposite sides of the circular cell, and for this to occur, the ants must align relative to each other.

### Pair correlation normalization

The pair correlation function in 2D far from system boundaries is $g\left(r\right)=\frac{1}{\rho }\frac{N\left(r\right)}{2\pi r\Delta r}$. Our confinement geometry requires us to use a normalization factor different from ${\left(2\pi r \right)}^{-1}$, which we call the available arc length, *L*. For two ants, labeled *X* and *Y*, with ant X a distance *R* from the center of the cell, the available arc length for ant *Y* to be found a distance *r* away from ant *X*, in a cell with diameter *D* is:

3 $$L\left(X,Y\right)=\left\{\begin{array}{cc}2r\left(\pi -{{\arccos }}\left(\frac{{D}^{2}-4{R}^{2}-4{r}^{2}}{8{Rr}}\right)\right),&R+r\, < \,\frac{D}{2}\\ 2\pi r,&R+r\le \frac{D}{2}\end{array}\right.$$

The available arc length $L\left(X,Y\right)\ne L(Y,X)$, because *R* depends only on the position of ant X, so *L* is calculated twice for each pair of ants, as demonstrated in Fig. 5b. From the measured positions of the ants, we calculate the center-to-center distances from each ant to each other ant and the available arc lengths for each of those distances every frame. We sort the measured available arc lengths in all trials with a given *N* by the pairs’ center-to-center distances into 0.14 mm bins, which results in at least 10^4^ counts in each bin for 0.3 cm < *r* < 7 cm.

Next, we calculate a weighted measure of the counts for each bin, $\omega \left(r\right)={\sum }_{i}{L}_{i}^{-1}$, where the summation runs over pairs in each bin. This calculation of *ω*(*r*) normalizes each count individually by its associated available arc length. For example, consider a pair of ants found at a distance apart in which only *π* radians are possible at that distance apart in the cell. Weighting this count by L^−1^ is equivalent to adding a phantom count to make up for the fact that this count was made despite it being half as statistically likely as it would be if the whole circle, 2*πr*, was available.

In a system without boundary concerns, *ω*(*r*) would simplify to $\omega \left(r\right)={\sum }_{i}1/(2\pi r)=N(r)/(2\pi r)$ where *N*(*r*) is the number of ants in the circular annulus that corresponds to the bin starting at *r*. Finally, we normalize *ω*(*r*) by its average value between 4 cm and 6 cm to insure that *g*(*r*) → 1 appropriately at long distances. Hence: $g\left(r\right)=\omega (r)/{\left\langle \omega \left(r\right)\right\rangle }_{4-6}$.

The results for various *N* are shown in Fig. 5c. The pair correlations show a strong peak around *r* = *l* and then decays to 1 at long distances. These pair correlations are used to calculate the PMF, ${U}_{p}=-{{{{{\rm{ln}}}}}}\left(g\left(r\right)\right)$, which are shown in Fig. 5d.

### Cluster detection

**Fig. 6:**
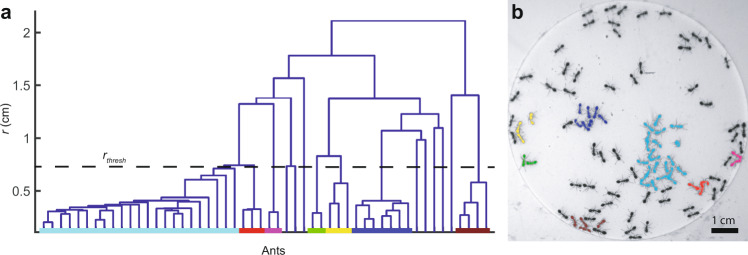
Cluster analysis. **a** Dendrogram for one frame of an experiment with *N* = 100 ants. Clusters of ants correspond to branches that cross the *r*_*thresh*_ line. Leaves that correspond to ants in a single cluster terminate on a line colored to match the cluster in Fig. 6b. Only clusters of more than 1 ant are shown. **b** Clusters of stationary ants divided using the previous dendrogram. The clusters have, in order of size, *N*_*c*_ = 22, 7, 4, 3, 3, 2, and 2 ants.

To characterize the clusters, we first filter out any ants that are moving in each frame with $\left|\vec{v}\left(t\right)\right| > 1\,{{{{{{\rm{mm}}}}}}}/{{{{{\rm{s}}}}}}$, so that we are left with only “stationary” ants in each frame. For example, only stationary ants are highlighted in Fig. 3a. Then, in each frame, we sort the stationary ants into clusters by creating a dendrogram, which is a common chart used in clustering analysis^46^, from their center-to-center-distances and choosing a cutoff distance that corresponds to about twice the length of an ant, as shown in Fig. 6a. We include in our cluster size distribution all observed clusters, including those that we only detect for a single frame.

The *x*-axis in Fig. 6a shows the labels associated to each stationary ant. Each ant starts out as a leaf on the dendrogram, and its branch continues vertically until it reaches a *y*-value that corresponds to the distance between the starting ant and its nearest neighbor, at which point the branch merges with the branch that includes its neighbor. Then, this merged branch, which now represents a cluster, continues vertically upwards until a *y*-value that corresponds to the minimum distance between any ant in this cluster and another ant outside of the cluster, and the branches merge again.

This continues until all the ants are included in one branch (the trunk) at the top. Notice that many of the leaves first join around *r* = *l*, consistent with the location of the local minima in Fig. 2 of the main text. Segregating the ants into individual clusters is a matter of picking a *r*_*thresh*_ checking which ants are included in each branch *r*_*thresh*_ intersects. Choosing *r*_*thresh*_ = 7 mm ≈ 2*l* results in the clusters designated by different colored lines on the *x*-axis in Fig. 6a. The ants in Fig. 6b, have been color coded according to the color of their leaf in Fig. 3a. The biggest cluster in this image contains *N*_*c*_ = 22 ants.

For our calculation of the radius of gyration, *R*_*g*_, for each cluster, we treat the masses of the ants as equal and treat each ant as a point particle located at its centroid so that ${R}_{g}^{2}=\frac{1}{{N}_{c}}{{\sum }_{i}({{{{{{\bf{r}}}}}}}_{i}-{{{{{{\bf{r}}}}}}}_{{CM}})}^{2}$, where **r**_*CM*_ is the average position of the ants in the cluster.

### Dynamics

**Fig. 7:**
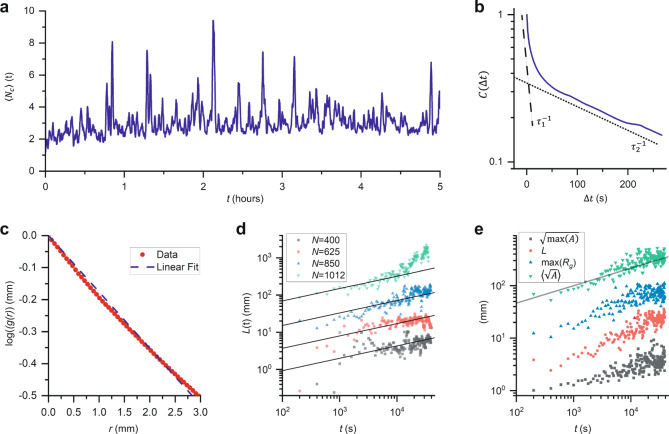
Cluster dynamics. **a** Average size of the clusters measured in the experiment with *N* = 40 ants. The cluster sizes continually fluctuate. **b** Autocorrelation function for an experiment with *N* = 625 ants (solid navy line). We measure two timescales from this function, *τ*_1_ (dashed line) and *τ*_2_ (dotted line). **c** The spatial autocorrelation function of pixels associated to the stationary phase as a function of time for an experiment with *N* = 625. The dashed line is the best linear fit of log(*g*(*r*)), and its slope corresponds to *−*1/*L*. **d** Correlation length scale for the clusters measured in experiments with *N* ≥ 400. The data for each experiment has been offset vertically and lines representing *L* ∝ *t*^1/3^ have been added for clarity. **e** Different measurements of relevant lengths in the experiment with *N* = 850: $\left\langle \sqrt{{A}_{i}}\right\rangle$ (gray squares), *L* (red circles), max(*R*_*g*_) (blue upwards triangles),$\sqrt{{{\max }}\left(A\right)}$ (green downwards triangles). The solid gray line is $L\propto {t}^{1/3}$.

In our tracking experiments at low density, we find the average cluster size does not continue to grow over the course of the experiment. For example, Fig. 7a shows the average size of the clusters over the course of a 5-h experiment, smoothed with a 1-minute rolling average. This stands in contrast to what has been seen in the aggregations of other species of ants^32^ and to the behavior of the phase-separated fire ants.

To begin measuring the dynamics of the clusters in experiments at higher ant densities, we divide the cell into 2 mm × 2 mm bins, measure the time autocorrelation function for these bins, and then average these autocorrelations together. We denote this autocorrelation function *C*(Δ*t*). Figure 7b is a typical example of the result. We always find two decays with characteristic timescales *τ*_1_ and *τ*_2_.

We find that *τ*_2_ is always on the order of several hundred seconds, which is approximately how long it takes for the clusters to “dissolve” and reform. In contrast, *τ*_1_ is on the order of ten seconds, which corresponds to about how long it takes an ant in the moving phase to move about an ant length l.

While the clusters in the phase-separated samples “evaporate” and reform, they also seem to coarsen. To measure the coarsening of the clusters during experiments with *N* ≥ 400, we first measure the pair correlation function for the pixels that correspond to the dense phase, like those shown in green in  9c. Let *ϕ*(*x*, *y*) represent the phase associated to each pixel, where *ϕ* = 1 corresponds to a pixel representing part of the dense phase and *ϕ* = 0 corresponds to a pixel that is not included in the dense phase. We calculate the spatial autocorrelation using the convolution theorem: $g\left(\Delta x,\,\Delta y\right)={{{{{{\mathcal{F}}}}}}}^{-1}\left({{{{{\mathcal{F}}}}}}\left(\phi \left(x,\,y\right)\right){{{{{{\mathcal{F}}}}}}}^{*}\left(\phi \left(x,\,y\right)\right)\right).$ Then we bin the 2D spatial autocorrelation by distance from (0,0), which we locate at the upper left corner of our images, and normalize the result by *g*(0,0) to obtain *g*(*r*). The pair correlation corresponding to the frame shown in Fig. 9c is shown in Fig. 7c. For small *r*, $g\left(r\right)\;\approx \;{{\exp }}\left(-\frac{r}{L}\right)$, where *L* is a constant length scale. We fit ln *g*(*r*) versus *r* to obtain *L*. We sample this length scale every 200 frames to examine the growth of the clusters over the course of the experiment. We find that the coarsening of the clusters is consistent with $L\propto {t}^{\frac{1}{3}}$, as shown in Fig. 7d, where the points for each trial have been offset vertically for clarity and the solid lines represent $L\propto {t}^{\frac{1}{3}}$; this ${t}^{1/3}$ scaling is typical of MIPS and of spinodal decomposition processes. Importantly, we find similar results for other possible measures of the characteristic length scale; see Fig. 7e, where we show the scaling of 〈$\sqrt{{A}_{i}}$〉, with *A*_*i*_ the area of the clusters (green), $\sqrt{{{\max }}(A)}$, with max(*A*) the area of the largest cluster (gray), and max(*R*_*g*_), the radius of gyration of the largest cluster (blue), each offset vertically for clarity. Notice that the scaling of each of these measures is approximately the same as the scaling of *L*(*t*) (red), which is $L\left(t\right)\propto {t}^{1/3}$ (black line).

### “Vapor pressure” related analysis

**Fig. 8:**
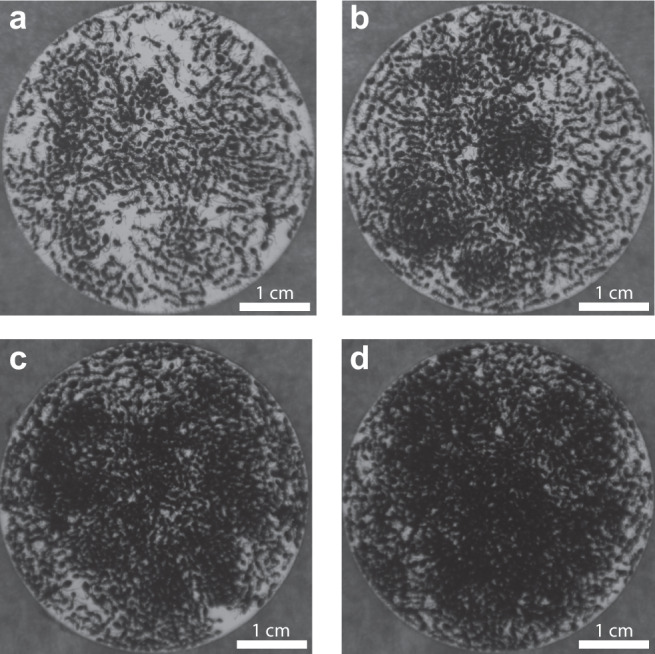
Effects of increasing density. **a**–**d** Frames from experiments using *N* = 438, 625, 850, and 1012 ants, respectively. Notice that both the clusters and the background of moving ants become denser when the number of ants in the cell increases.

The density of both phases clearly increases with the global density, as shown in Fig. 8a–d, which shows trials with *N* = 438, 625, 850, and 1012 ants, respectively. To quantify this and generate the plot in Fig. 4a, we measure the intensity of light extinguished by the two phases.

We process the video to calculate the background intensity of light, which is the intensity we would measure if there were no ants in the cell, by taking the maximum intensity recorded for each pixel over the course of the 5 h experiment, ${{{{{\rm{BG}}}}}}(x,\,y)=\mathop{{{\max }}}\limits_{t}I(x,\,y,\,t)$.

**Fig. 9:**
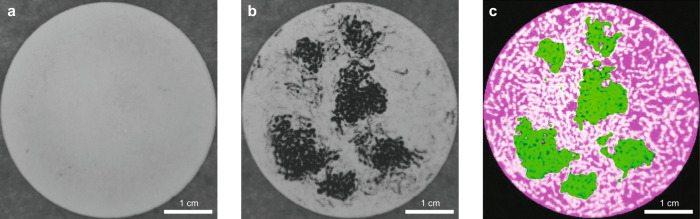
Density analysis. **a** The maximum projection for each pixel over the course of an experiment with *N* = 625 ants. **b** The maximum projection for each pixel in the 30 s following the image shown in Fig. 8b. **c** A composite image showing the results of blurring and thresholding the difference between the previous two panels to isolate the moving (magenta) and stationary (green) phases. These phases are overlayed onto the inverted raw image.

Because this observation window is much longer than the timescales associated with either phase, the background calculation effectively captures an empty cell, as shown in Fig. 9a.

To detect the stationary clusters, but not the moving ants, we repeat this basic process with 30 s windows. Because this is in between the timescales we associate with the two phases, *τ*_1_ and *τ*_2_, we now detect the stationary clusters, as shown in Fig. 9c. Subtracting this temporary maximum from the background, $A\left(x,\,y,\,t^{\prime} \right)={BG}(x,\,y)-\mathop{{{\max }}}\limits_{t}I(x,\,y,\,{t}^{{\prime} }+t)$, with *t*=30 s, yields the extinction of light by the clusters. We blur this result with a circular pillbox averaging filter with a diameter of 1.2 mm and choose a threshold to detect the regions that we associate with the clusters, as shown in the green overlay in Fig. 9c. We associate the rest of the cell with the moving phase, shown with the magenta overlay in Fig. 9c. Our results are not sensitive to reasonable choices of threshold.

Finally, we take the average value of the extinction intensity separately in each of the two regions and report them in Fig. 4a.

### Statistics

All videos of the ants were captured at 3.75 fps and pair distances, velocities, available arc lengths, and cluster sizes were calculated for every ant or pair of ants in each frame. Because there are $\left({N}\atop{2}\right)$ pairs in each frame, many fewer frames are necessary to calculate *g*(*r*) and *v*(*r*) in experiments with more ants. Table 1 shows the number of trials, hours, and measured pairs we observed in experiments with *N* ≤ 100.

**Table 1:** Statistical details

*N*	Trials	Trial length (hours)	total hours	total pairs
1	15	3	45	0
2	22	3	65	8.78E+05
3	13	3	39	1.58E+06
5	7	3	21	2.84E+06
10	2	3	6	3.65E+06
30	3	5	15	8.81E+07
40	1	5	5	5.27E+07
100	1	5	5	3.34E+08

The number of trials conducted using varying numbers of ants, the length of the trials in hours, total hours of observed ant behavior, and the number of pairs used to calculate *g*(*r*) and *v*(*r*).

For each of the trials with *N* ≥ 400, we performed each trial once for 3 h. The points we reported in Fig. 4a correspond to the densities we measured in each phase 0.75, 1.5, and 2.25 h into each of the experiments.

### Reporting summary

Further information on research design is available in the Nature Research Reporting Summary linked to this article.

## Supplementary information

Descriptions of additional supplementary files

Supplementary Movie 1

Supplementary Movie 2

Supplementary Software

Reporting Summary

## Data Availability

The data of this study are provided as Source data. Source data are provided with this paper.

## Source data

Source Data

## Code availability

The codes used to analyze the data are provided as Source Software.
