# Supplementary material for: Social interactions lead to motility-induced phase separation in fire ants
Source: Nat Commun. 2022 Nov 7;13:6710. doi: 10.1038/s41467-022-34181-0 (PMC9640710; doi:10.1038/s41467-022-34181-0)
Supplement: Supplementary file 1 — Descriptions of additional supplementary files [file 41467_2022_34181_MOESM1_ESM.pdf]

Description of additional supplementary files

Supplementary Movie 1: A D = 9.00 cm cell containing N = 100 fire ants showing the formation and break up of short-lived clusters of stationary ants. The video plays at 20 × the speed at which it was recorded.

Supplementary Movie 2: A D = 4.50 cm cell containing N ≈ 625 fire ants. The ants form dynamic heterogeneities so that there are dense clusters of stationary ants surrounded by a background of actively moving ants. The video plays at 50 × the speed at which it was recorded.

Supplementary Software:

“autotracker.mat” – algorithm used to detect the raw ants in images, track them, and then calculate their frame-by-frame correlations.

“lengthscale.mat” – algorithm used to calculate the average size of the clusters in the high-density experiment.

“two_densities.mat” – algorithm used to detect the average densities of each of the two phases.
