# Supplementary material for: Social interactions lead to motility-induced phase separation in fire ants
Source: Nat Commun. 2022 Nov 7;13:6710. doi: 10.1038/s41467-022-34181-0 (PMC9640710; doi:10.1038/s41467-022-34181-0)
Supplement: Supplementary file 5 — Reporting Summary [file 41467_2022_34181_MOESM5_ESM.pdf]

Corresponding author(s): Alberto Fernandez-Nieves

Last updated by author(s): Apr 26, 2022

Reporting Summary

Nature Portfolio wishes to improve the reproducibility of the work that we publish. This form provides structure for consistency and transparency in reporting. For further information on Nature Portfolio policies, see our Editorial Policies and the Editorial Policy Checklist.

Statistics

For all statistical analyses, confirm that the following items are present in the figure legend, table legend, main text, or Methods section.

n/a	Confirmed	
☐	☒	The exact sample size (n) for each experimental group/condition, given as a discrete number and unit of measurement
☐	☒	A statement on whether measurements were taken from distinct samples or whether the same sample was measured repeatedly
☒	☐	The statistical test(s) used AND whether they are one- or two-sided<br>*Only common tests should be described solely by name; describe more complex techniques in the Methods section.*
☒	☐	A description of all covariates tested
☒	☐	A description of any assumptions or corrections, such as tests of normality and adjustment for multiple comparisons
☐	☒	A full description of the statistical parameters including central tendency (e.g. means) or other basic estimates (e.g. regression coefficient) AND variation (e.g. standard deviation) or associated estimates of uncertainty (e.g. confidence intervals)
☒	☐	For null hypothesis testing, the test statistic (e.g. F, t, r) with confidence intervals, effect sizes, degrees of freedom and P value noted<br>*Give P values as exact values whenever suitable.*
☒	☐	For Bayesian analysis, information on the choice of priors and Markov chain Monte Carlo settings
☒	☐	For hierarchical and complex designs, identification of the appropriate level for tests and full reporting of outcomes
☒	☐	Estimates of effect sizes (e.g. Cohen's d, Pearson's r), indicating how they were calculated

Our web collection on statistics for biologists contains articles on many of the points above.

Software and code

Policy information about availability of computer code

Data collection	Images were collected using CCD software
Data analysis	Data analysis was performed with software developed in our lab

For manuscripts utilizing custom algorithms or software that are central to the research but not yet described in published literature, software must be made available to editors and reviewers. We strongly encourage code deposition in a community repository (e.g. GitHub). See the Nature Portfolio guidelines for submitting code & software for further information.

Data

Policy information about availability of data

All manuscripts must include a data availability statement. This statement should provide the following information, where applicable:

- Accession codes, unique identifiers, or web links for publicly available datasets
- A description of any restrictions on data availability
- For clinical datasets or third party data, please ensure that the statement adheres to our policy

All data will be made available upon reasonable requests to the authors

Field-specific reporting

Please select the one below that is the best fit for your research. If you are not sure, read the appropriate sections before making your selection.

☐ Life sciences ☐ Behavioural & social sciences ☒ Ecological, evolutionary & environmental sciences

For a reference copy of the document with all sections, see nature.com/documents/nr-reporting-summary-flat.pdf

Ecological, evolutionary & environmental sciences study design

All studies must disclose on these points even when the disclosure is negative.

Study description	We filmed and tracked groups of various numbers of fire ants in a small enclosure over a series of 68 3- and 5-hour experiments. Details on the breakdown of the number of ants used in each trial and our rationale are given in the "Statistics" section of our SI.
Research sample	Large groups of fire ants, Solenopsis Invicta, were collected from a location in Kennesaw, GA (34°01'10.7"N 84°31'35.6"W). After collection, smaller samples of ants were selected from these large groups over the next 4 weeks.
Sampling strategy	We continued taking samples until we had obtained at least 10,000 counts of ants in each of our bins. The ants were binned by pair separation into 0.1 mm bins Once this condition was reached, we continued performing experiments until our U_p curves were smooth.
Data collection	We filmed the ants from above at a rate of 3.75 fps, and used this information to extract the positions and orientations or optical density of the ants over time.
Timing and spatial scale	Tracking experiments were performed over several years, beginning in November 2016 and ending in Feb 2020. Experiments with dense ant collectives, in which tracking was impossible, were performed in August and September 2020.
Data exclusions	No data was excluded.
Reproducibility	The large groups of fire ants in our lab were all provided with the same care in our lab, including constant availability of the same food, availability of water, and exposure to light. All attempts to repeat the experiment resulted in reproducible data.
Randomization	For the purpose of our tracking algorithm, we tended to collect ants from the nest that were roughly the same size (all small, all medium, or all large). We randomly selected more ants than needed for each trial and then removed ants that were furthest from the median size until we were left with the correct number of ants.
Blinding	We do not think blinding was relevant to our experiment because we did not have information to give the ants that could bias their behavior one way or the other.

Did the study involve field work? ☒ Yes ☐ No

Field work, collection and transport

Field conditions	We collected the ants the day(s) after rainfall and dug out the whole colony.
Location	Kennesaw, GA (34°01'10.7"N 84°31'35.6"W)
Access & import/export	Fire ants can be found in Georgia. We accessed the fields by car and dug up the ant colony from the ground usually after rain
Disturbance	No disturbance was caused

Reporting for specific materials, systems and methods

We require information from authors about some types of materials, experimental systems and methods used in many studies. Here, indicate whether each material, system or method listed is relevant to your study. If you are not sure if a list item applies to your research, read the appropriate section before selecting a response.

Materials & experimental systems

n/a	Involved in the study
☒	☐ Antibodies
☒	☐ Eukaryotic cell lines
☒	☐ Palaeontology and archaeology
☐	☒ Animals and other organisms
☒	☐ Human research participants
☒	☐ Clinical data
☒	☐ Dual use research of concern

Methods

n/a	Involved in the study
☒	☐ ChIP-seq
☒	☐ Flow cytometry
☒	☐ MRI-based neuroimaging

Animals and other organisms

Policy information about studies involving animals; ARRIVE guidelines recommended for reporting animal research

Laboratory animals	none
Wild animals	Fire ants, Solenopsis Invicta
Field-collected samples	We kept the ants in bins with water and a protein source
Ethics oversight	No ethics oversight is required to work with ants in Georgia, USA

Note that full information on the approval of the study protocol must also be provided in the manuscript.
